# Correction: Cytotoxic trichothecene-type sesquiterpenes from the sponge-derived fungus *Stachybotrys chartarum* with tyrosine kinase inhibition

**DOI:** 10.1039/c9ra90052g

**Published:** 2019-07-10

**Authors:** Yong Li, Dong Liu, Zhongbin Cheng, Peter Proksch, Wenhan Lin

**Affiliations:** State Key Laboratory of Natural and Biomimetic Drugs, Peking University Beijing 100191 P. R. China whlin@bjmu.edu.cn +86-10-82806188; Institute of Pharmaceutical Biology and Biotechnology, Heinrich-Heine University 40225 Duesseldorf Germany

## Abstract

Correction for ‘Cytotoxic trichothecene-type sesquiterpenes from the sponge-derived fungus *Stachybotrys chartarum* with tyrosine kinase inhibition’ by Yong Li *et al.*, *RSC Adv.*, 2017, **7**, 7259–7267.

The authors regret that compound 5 was incorrectly labelled in Fig. 4 in the original manuscript. The corrected figure is shown below.

**Fig. 4:**
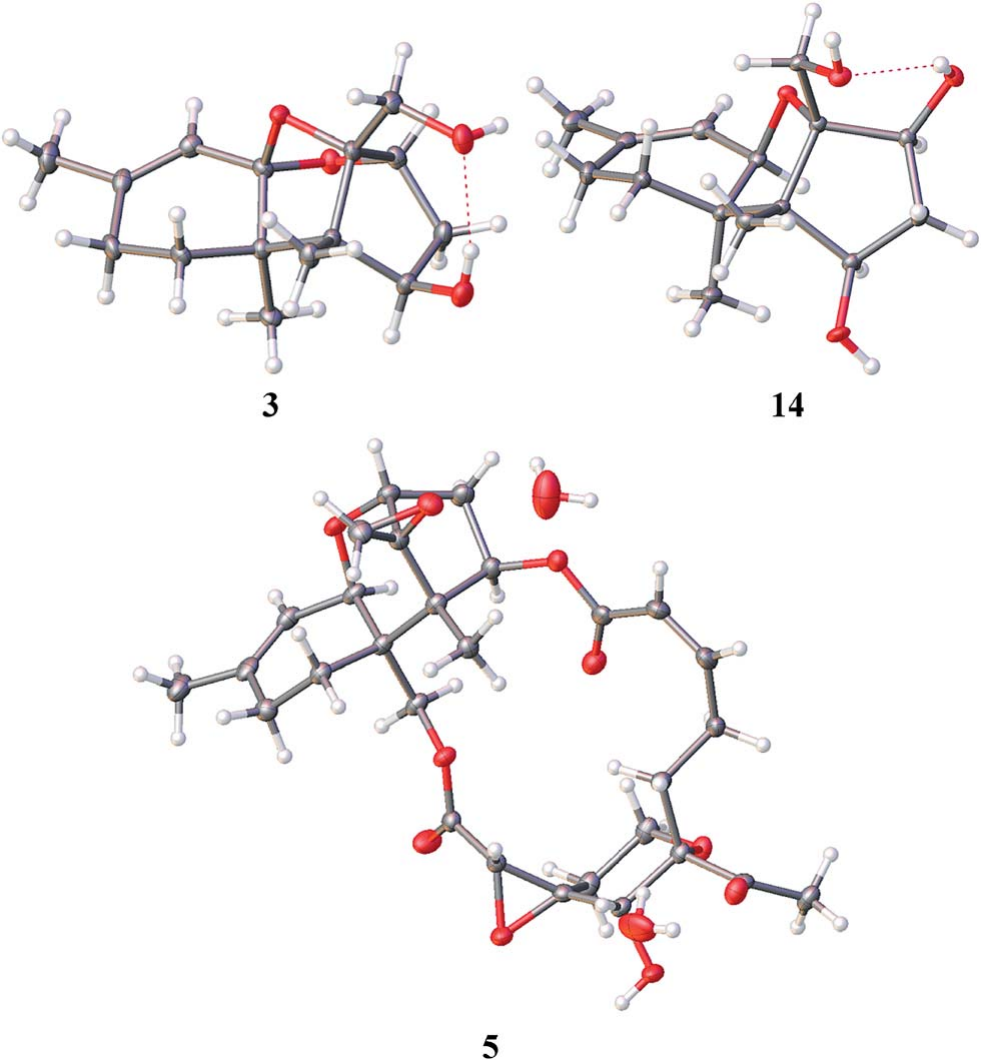
X-ray structures of 3, 5, and 14.

In addition, on page 7462 the sentence “In the present work, the absolute configurations of satratoxin G (6) and 2,4,12-trihydroxyapotrichothecene (14) were established by the analyses of the X-ray diffraction data using Flack parameters (Fig. 4)” is corrected to “In the present work, the absolute configurations of mytoxin A (5) and 2,4,12-trihydroxyapotrichothecene (14) were established by the analyses of the X-ray diffraction data using Flack parameters (Fig. 4)”.

The Royal Society of Chemistry apologises for these errors and any consequent inconvenience to authors and readers.

## Supplementary Material

